# 5-Hydroxymethylfurfural: A Particularly Harmful Molecule Inducing Toxic Lipids and Proteins?

**DOI:** 10.3390/molecules30193897

**Published:** 2025-09-26

**Authors:** Joachim Greilberger, Georg Feigl, Matthias Greilberger, Simona Bystrianska, Michaela Greilberger

**Affiliations:** 1 Division of Medicinal Chemistry, Otto Loewi Research Center, Medical University of Graz, 8036 Graz, Austria; 2 Institute of Anatomy and Clinical Morphology, Witten University, 58448 Witten, Germany; georg.feigl@uni-wh.de; 3 Institut fuer Laborwissenschaften Dr. Greilberger, Schwarzl Klinik, 8301 Lassnitzhoehe, Austria; greilberger.matthias@gmail.com (M.G.); institut@laborwissenschaft.at (M.G.); 4 Zentrum für Regenerative Medizin des Bewegungsapparates, 4040 Linz, Austria; ordination@bystrianska.at

**Keywords:** vitamin C (VitC), 5-Hydroxy-methyl-furfural (5-HMF), Malondialdehyde (MDA), 4-Hydroxynonenal (HNE), thiobarbituric acid reacting substances (TBARS), Fenton reaction, lipid peroxidation, atherosclerosis

## Abstract

**Introduction:** 5-HMF is a molecule found in carbohydrate-rich foods that is associated not only with cancer and anaphylactic reactions, but also with anti-oxidant properties. Questions arose as to whether 5-HMF exhibited a catalytic effect in relation to lipid peroxidation and lipoprotein oxidation in presence of metals and/or radicals. **Methods:** Peroxynitrite (ONOO^−^)-induced chemiluminescence and ONOO^−^ nitration of tyrosine residues on BSA using anti-nitro-tyrosine-antibodies were used to measure the protection of 5-HMF against peroxides or nitration compared to vitamin C (VitC). The reductive potential of 5-HMF or VitC on Cu^2+^ or Fe^3^ was estimated using the bicinchoninic acid (BCA) or Fenton-complex method. Human plasma was used to measure the generation of malondialdehyde (MDA), 4-hydroxynonenal (HNE), and total thiols after Fe^2+^/H_2_O_2_ oxidation in the presence of different concentrations of 5-HMF or VitC. Finally, Cu^2+^ oxidation of LDL after 4 h was carried out with 5-HMF or VitC, measuring the concentration of MDA in LDL with the thiobarbituric assay (TBARS). **Results:** VitC was 4-fold more effective than 5-HMF in scavenging ONOO^−^ to nearly 91.5% at 4 mM, with the exception of 0.16 mM, where the reduction of ONOO^−^ by VitC was 3.3-fold weaker compared to 0.16 mM 5-HMF. VitC or 5-HMF at a concentration of 6 mM inhibited the nitration of tyrosine residues on BSA to nearly 90% with a similar course. While 5-HMF reduced free Fe^3+^ in presence of phenanthroline, forming Fe^2+^ (phenantroleine)_3_ [Fe^2+^(phe)_3_] or complexed Cu^2+^(BCA)_4_ to Cu^+^(BCA)_4_ weakly, VitC was 7- to 19-fold effective in doing so over all the used concentrations (0–25 mM). A Fe^2+^—H_2_O_2_ solution mixed with human plasma showed a 6–10 times higher optical density (OD) of MDA or HNE in the presence of 5-HMF compared to VitC. The level of thiols was significantly decreased in the presence of higher VitC levels (1 mM: 198.4 ± 7.7 µM; 2 mM: 160.0 ± 13.4 µM) compared to equal 5-HMF amounts (2562 ± 7.8 µM or 242.4 ± 2.5 µM), whereas the usage of lower levels at 0.25 µM 5-HMF resulted in a significant decrease in thiols (272.4 ± 4.0 µM) compared to VitC (312.3 ± 19.7 µM). Both VitC and 5-HMF accelerated copper-mediated oxidation of LDL equally: while the TBARS levels from 4 h oxidized LDL reached 137.7 ± 12.3 nmol/mg, it was 1.7-fold higher using 6 mM VitC (259.9 ± 10.4 nmol/mg) or 6 mM 5-HMF (239.3 ± 10.2 nmol/mg). **Conclusions:** 5-HMF appeared to have more pro-oxidative potential compared to VitC by causing lipid peroxidation as well as protein oxidation.

## 1. Introduction

5-HMF is a product that occurs naturally on the one hand [1,2], but on the other hand, by the thermal treatment of foodstuffs using the so-called Maillard reaction, especially in sugar-containing solutions for the production of sugar liqueur or caramel liqueur, which are used as flavorings in the food industry. The daily intake of 5-HMF by humans has been calculated to be approximately 4 to 30 mg, but this can be significantly increased to 350 mg per day through the consumption of 5-HMF from prunes or prune-based drinks, which are particularly rich in 5-HMF (e.g., 707 mg/kg in prunes and 1022 mg/kg in prune drinks). However, it has also been shown that cocoa-containing beverages had a 5-HMF content of up to 503 mg/kg. Sugar liqueur and smoke flavorings showed particularly high 5-HMF values of up to 27,300 mg/kg according to the EFSA report [3]. The importance of a risk assessment of 5-HMF has been fueled by its possible or potential carcinogenicity, which has not yet been assessed due to the limited data available. No toxic effects were observed in animal experiments with a daily dose of 5-HMF in the range of 80–100 mg/kg body weight. Nevertheless, it should be noted that 5-HMF or its metabolite 5-SMF might be carcinogenic in studies in mice [4,5]. Unfortunately, 5-HMF seems to possess a potential risk to human and animal health, although cellular studies on cancer cells have shown an anti-carcinogenic and anti-anti-oxidative effect in combination with alpha-ketoglutaric acid, as seen in Jurkat cell lines [6].

Besides its anti-sickling properties [7], it was reported that 5-HMF increased in mucosal gastric epithelial cells reactive oxygen species (ROS), which led them to apoptosis [8]. It has been shown that 5-HMF in larger quantities increases the death of heart cells by having a sensitive negative effect on the pericardial edema ratio and the heart rate [9]. This led to a developmental disorder of the cardiovascular vessels and thus also to altered gene expression. It is strongly suspected that 5-HMF, through oxidative stress, disrupts glucose and lipid metabolism, which directly leads to intestinal damage and ultimately impairs the development of Drosophila melanogaster [10]. But it was also discussed that 5-HMF was able to positively influence the anti-inflammatory effect in the LPS-induced inflammatory response by inhibiting the MAPK, NF-κB, and Akt/mTOR signaling pathways [11].

Studies on erythrocytes showed an increase in the enzyme activity of anti-oxidant-acting enzymes such as superoxide dismutase, catalase, and glutathione peroxidase due to 5-HMF, whereby 5-HMF reduced AAPH radical-induced hemolysis in a concentration-dependent manner [12].

With regard to the oxidation of human plasma by a Fenton reaction (Fe^2+^/H_2_O_2_), no data has been collected to date on the efficacy of 5-HMF, nor has any data been collected on copper-mediated lipid peroxidation of lipoproteins, e.g., LDL, in presence of 5-HMF. In the literature, no data were available about its reductive or oxidative potential with metal ions, like Cu^2+^ or Fe^3+^, or with Fe^2+^/H_2_O_2_. The basis of all oxidative and anti-oxidative properties of a substance is to compare it with a similar and known substance found in nature and presented in fresh fruit and vegetables, like vitamin C (VitC).

Besides the powerful anti-oxidative effects of VitC, it was reported that under special circumstances, VitC possesses pro-oxidative effects, e.g., during the Fenton reaction (Fe^2+^/H_2_O_2_) in generating hydroxyl radicals. During this mechanism, Fe^2+^ is oxidized to Fe^3+^, but in presence of VitC, it can be reduced to Fe^2+^ again, catalyzing the reaction to generate additional toxic hydroxyl radicals [13].

Currently, nitric oxide (NO*) is the only known molecule in vivo that is produced in sufficiently high concentrations and can react quickly enough with superoxide anion radicals (O_2_*^−^) [14] to peroxides because O_2_*^−^ can no longer be broken down by superoxide dismutase (SOD) due to increased amounts of O_2_*^−^. As a representative of peroxides, ONOO^−^ is capable of nitrating and oxidizing natural compounds. A classic example is the nitration of tyrosine to nitro-tyrosine, e.g., on proteins such as albumin.

The literature already indicated that 5-HMF was able to reduce peroxynitrite and that the nitration of tyrosine residues on albumin was effectively prevented [15]. However, a comparison of 5-HMF alone with VitC, currently one of the most potent anti-oxidants against peroxynitrite, is still lacking.

Following parameters are used to estimate the anti- or pro-oxidative behavior of 5-HMF by the determination of (i) its properties to reduce Cu^2+^(BCA)_4_ to Cu^+^(BCA)_4_ and to reduce Fe^3+^ in a phenanthroline containing solution forming a Fe^2+^(phe)_3_ complex (Ferroin) in comparison to VitC, (ii) its scavenging effect with peroxynitrite vs. VitC, (iii) its inhibiting effect in the generation of nitro-tyrosine residues in BSA in presence of peroxynitrite compared to VitC, (iv) its influence during Fe^2+^/H_2_O_2_ oxidation of human plasma measuring malondialdehyde (MDA), 4-hydroxy-nonenal (HNE) and total thiol content compared to VitC, (v) and its influence in the generation of lipid peroxides during copper mediated oxidation of LDL with the estimation of thiobarbituric acid reacting substances (TBARS) vs. VitC [10].

## 2. Results

### 2.1. ONOO^−^—Luminol Measurements Using 5-HMF vs. VitC

**Figure 1:**
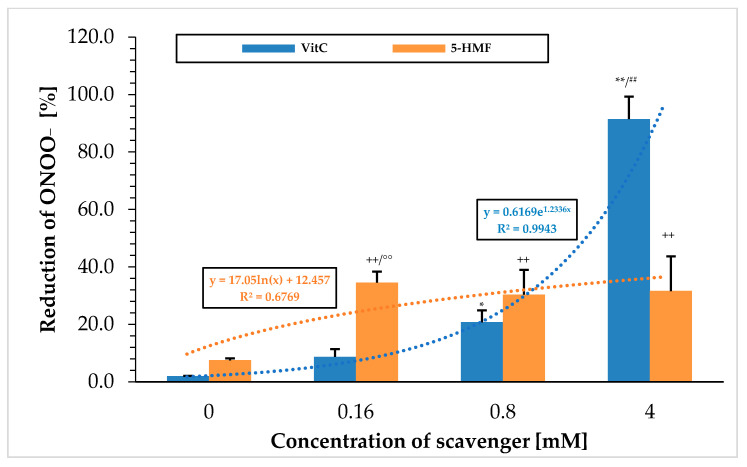
Comparison of different 5-HMF and VitC concentrations in reducing peroxynitrite chemiluminescence signal. Zero concentrations were set to 0% for each substance. * *p* < 0.01: significance between 0.16 mM VitC and 0 mM VitC; ** *p* < 0.001: significance between 0.8 and 4 mM VitC; ^++^ *p* < 0.01: significance between 0 mM 5-HMF and 0.16, 0.8 and 4 mM 5-HMF; °° *p* < 0.001: significance between 0.16 mM VitC and 0.16 mM 5-HMF; ^##^ *p* < 0.001: significance and between 4 mM VitC and 4 mM 5-HMF.

Figure 1 shows the ability to break down peroxynitrite using the chemiluminescence technique with the two substances VitC and 5-HMF. VitC showed a steadily increasing percentage value in scavenging ONOO^−^ with increasing concentrations. When using 0.16 mM VitC, the signal was significantly increased from the initial value (0 mM VitC; 2.0 ± 0.1%; *n* = 3) to 8.7 ± 2.7% (*p* < 0.05) and, at a VitC concentration of 0.8 mM, even highly significantly (20.7 ± 4.2%; *n* = 3; *p* < 0.001). The greatest reduction in the peroxynitrite signal was achieved at a VitC concentration of 4 mM with 91.5 ± 7.8%, which was significantly higher compared to 0.8 mM VitC (*p* < 0.001). Correlating the concentration of used VitC with the ONOO^−^ reducing effect revealed an exponential relationship. This means that VitC had a smaller effect at low concentration ranges but was very effective at high concentrations. 5-HMF showed a different pattern: the greatest reduction of ONOO^−^ in the initial signal was already achieved using a 0.16 mM 5-HMF concentration (34.5 ± 3.9%), which was significantly higher compared to 0 mM 5-HMF (7.5 ± 0.7%; *p* < 0.001). No further reduction in peroxynitrite was achieved at higher concentrations of 5-HMF: 0.8 mM 5-HMF (30.4 ± 8.6%) and 4 mM 5-HMF (31.6 ± 12.1%). Correlation of 5-HMF concentrations with the radical scavenging reduction of ONOO^−^ resulted in a logarithmic function, in which low concentration of 5-HMF showed a high reduction potential of ONOO^−^ but reached saturation quickly. Overall, 5-HMF had a small ONOO^−^ reduction potential of 1/3 of the output signal between 0.16 and 4 mM.

This was particularly evident in the comparison of VitC and 5-HMF at 0.16 mM, where 5-HMF was found to have a better ONOO^−^ scavenging effect (4-fold). At higher concentrations, this reducing property of ONOO^−^ changed: VitC was able to reduce almost all of the used ONOO^−^ when the concentration was increased up to 4 mM. At a concentration of 4 mM, the ONOO^−^ reduction potential for VitC was significantly 3-fold higher than for 5-HMF.

### 2.2. Estimation of Nitrated Tyrosine Residues on BSA in Presence of 5-HMF vs. VitC

**Figure 2:**
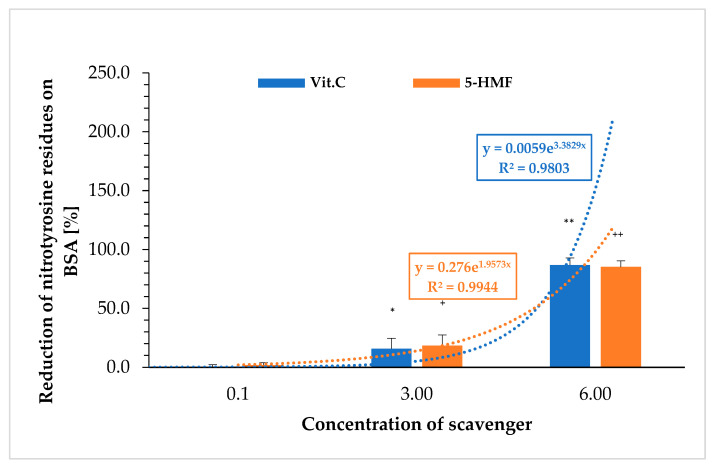
Comparison of different 5-HMF and VitC concentrations in reducing nitro-tyrosine signal on BSA. Zero concentrations were set to 0% for each substance. ^+^ *p* < 0.05: significance between 0 and 3 mM VitC; ^++^ *p* < 0.001: significance between 3 and 6 mM VitC. * *p* < 0.05: significance between 0 and 3 mM 5-HMF; ** *p* < 0.001: significance between 3 and 6 mM 5-HMF.

Figure 2 shows the extent to which 5-HMF and VitC prevent the nitration to nitro-tyrosine on BSA. The nitration of BSA was carried out with peroxynitrite and determined using an ELISA. The results were again calculated as a percentage of the respective initial signal (without VitC or 5-HMF).

Nitration of tyrosine on BSA was significantly protected by 3 mM VitC compared to the initial value (0 mM VitC: 0.1 ± 2.1%; 3 mM VitC: 15.7 ± 8.9%; *n* = 3; *p* < 0.05) and 6 mM (86.8 ± 6.2%; *n* = 3; *p* < 0.001). The same was observed using 5-HMF (0 mM 5-HMF: 0.0 ± 2.2%; 3 mM 5-HMF: 18.3 ± 9.1%; *n* = 3; *p* < 0.05) and 6 mM 5-HMF: 85.2 ± 5.2%; *n* = 3; *p* < 0.001). The correlation between concentration and reduction of nitration of tyrosine residues on BSA showed an exponential curve for both substances. No significant difference between VitC and 5-HMF was obtained comparing same concentrations with the reduction of nitrated tyrosine residues on BSA.

### 2.3. Estimation of the Reduction Potential of 5-HMF vs. VitC on Cu^+^(BCA)_4_

Table 1 shows the ability of VitC versus 5-HMF to reduce Cu^2+^(BCA)_4_ to a Cu^+^(BCA)_4_ complex. In general, 5-HMF showed a weaker property to reduce the copper in the complex compared to VitC. Correlation of the VitC concentrations with an estimated absorbance at 572 nm resulted in an exponential function (y = 7.5642^e−0.596x^) with a regression term of r = 0.99.

At a concentration of 16 µM, the Cu^+^(BCA)_4_ complex formation was more than 3-fold higher with VitC (0.377 ± 0.015; *n* = 3) compared to 5-HMF (0.102 ± 0.01; *n* = 3); using 31 µM, the reductive effect of VitC (0.711 ± 0.035; *n* = 3) was more than 6-fold higher compared to 5-HMF (0.116 ± 0.03; *n* = 3); and using 63 µM, a ten-fold higher reduction was estimated in favor of VitC (1.241 ± 0.064; *n* = 3) vs. 5-HMF (0.121 ± 0.01; *n* = 3). The highest used concentration of 125 µM showed a 19-fold higher absorbance using VitC compared to 5-HMF.

### 2.4. Generation of a Fe^2+^ Phenanthroline Complex [Fe^2+^(phe)_3_] by the Reduction of FeCl_3_ with 5-HMF or VitC

Table 2 shows the formation of a Fe^2+^ phenanthroline complex after the reduction of a Fe^3+^ chloride solution in the presence of VitC and 5-HMF.

A linear function was obtained correlating the VitC concentration with the estimated OD at 510 nm (y = −0.2277x + 1.0788) and a regression term of r = 0.99. Analog to the results obtained with the BCA method, 5-HMF showed a weaker property to reduce Fe^3+^ to Fe^2+^, forming a complex with phenanthroline compared to VitC.

At a concentration of 30 µM, the OD of the Fe^2+^(phe)_3_ complex was 2-fold higher in presence of VitC (0.199 ± 0.041; *n* = 3) compared to 5-HMF (0.098 ± 0.024; *n* = 3); using 50 µM, the reductive effect of VitC by measuring the OD (0.369 ± 0.035; *n* = 3) was 3.5-fold higher compared to 5-HMF (0.102 ± 0.016; *n* = 3); and using 100 µM, a 5-fold higher reduction in the OD was estimated in favor of VitC (0.581 ± 0.052; *n* = 3) vs. 5-HMF (0.118 ± 0.027; *n* = 3). An 8-fold higher reduction potential was obtained using 200 µM VitC (OD: 0.888 ± 0.021; *n* = 3) compared to 5-HMF (0.127 ± 0.022; *n* = 3).

### 2.5. Fenton Reaction of Human Plasma in Presence or Absence of 5-HMF Versus VitC Using Ferrum and H_2_O_2_

Fenton mixture (Fe^2+^/H_2_O_2_) was mixed with human plasma in absence or presence of different concentrations of VitC and 5-HMF and incubated for 90 min. at 37 °C. MDA (Table 3), HNE (Table 4), and the total thiols (Table 5) were then determined.

MDA showed in Table 3 a steady but weak increase using VitC concentrations between 0 and 1 mM. When 0.25 mM VitC was used, the OD achieved 0.099 ± 0.004 (*n* = 3), which differed significantly from the initial value (0 mM: 0.066 ± 0.002; *n* = 3; *p* < 0.05). The OD value of 0.5 mM vitamin C solution was 0.135 ± 0.009 (*n* = 3) and thus slightly significantly higher than the previous OD value with 0.25 mM vitamin C solution (*p* < 0.05). Finally, when a 1 mM VitC solution was used, the OD of MDA was highest with 0.192 ± 0.035 (*n* = 3).

Even at 0.125 mM, 5-HMF showed a 10-fold increase in the OD of 0.868 ± 0.017 (*n* = 3) with a high significance compared to 0 mM 5-HMF (0.072 ± 0.002; *p* < 0.001). The OD increased to 0.939 ± 0.024 (*n* = 3) using 0.25 mM 5-HMF significantly compared to the previous OD value (*p* < 0.01); using 0.5 mM 5-HMF, the OD increased to 1.098 ± 0.033 (*n* = 3) significantly (*p* < 0.001) compared to 0.25 mM 5-HMF, and finally, the usage of 1 mM 5-HMF resulted in the highest OD value of 1.209 ± 0.043 (*n* = 3; *p* < 0.001).

All the 5-HMF measurements were at least 8–10 times higher in the OD than the adequate VitC solutions and thus highly significant (*p* < 0.001).

In the presence of 0–1 mM VitC, there was a slight but steady increase in the OD of HNE at 586 nm s shown in Table 4. When using 0.5 mM VitC, the OD of HNE was 0.077 ± 0.008 (*n* = 3), which differed significantly from the baseline OD value (0 mM: 0.037 ± 0.001; *n* = 3; *p* < 0.05). The OD of HNE using a 1 mM VitC solution was 0.123 ± 0.034 (*n* = 3) and thus significantly (*p* < 0.01) higher than the baseline OD value and the previously used concentration of 0.5 mM VitC.

When using the 0.125 mM solution, 5-HMF showed a 15-fold increase in the OD from 0.609 ± 0.002 (*n* = 3) with a high significance compared to the 0 mM OD value (0.040 ± 0.001; *p* < 0.001). As the amount of 5-HMF raised, the OD increased steadily: 0.25 mM 5-HMF resulted in an OD value of 0.646 ± 0.020 (*n* = 3); 0.5 mM 5-HMF resulted in an OD of 0.747 ± 0.027 (*n* = 3) significantly (*p* < 0.01) compared to the previous 5-HMF value (0.25 mM); and finally, at 1 mM 5-HMF, the highest OD value of 0.783 ± 0.014 (*n* = 3; *p* < 0.001) was detected with a high significance compared to the OD with 0.5 mM 5-HMF.

All the 5-HMF measurements were at least 6 to 16 times higher than the adequate VitC solutions and thus highly significant (*p* < 0.001).

Table 5 shows the measurement of the total thiols in the plasmas oxidized with Fe^2+^ and H_2_O_2_ in the presence of different concentrations of VitC and 5-HMF. The zero value serves as the 100% baseline value.

Correlation of different VitC concentrations with estimated thiol concentrations resulted in a linear function (y = −47.51x + 401.35) as shown in Table 5. The regression value was obtained with r = 0.98. 0.25 mM VitC (312.3 ± 19.7 µM; *n* = 3) which achieved a significant reduction from the baseline value (0 mM: 340.8 ± 14.4 µM; *n* = 3; *p* < 0.01), an increase in the VitC concentration to 1 mM VitC (198.4 ± 7.7 µM; *n* = 3; *p* < 0.001) again resulted in a significant reduction in thiols to the 0.25 mM VitC value, and the highest used VitC concentration of 2 mM (160.0 ± 13.4 µM; *n* = 3; *p* < 0.01) showed a further reduction in thiols compared to the previous value of 1 mM VitC.

The use of 0.25 mM 5-HMF already showed a reduction in thiols of approximately 272.4 ± 4.0 µM (*n = 3*), which was significant with *p* < 0.001 compared to the baseline value (350.4 ± 36.6 µM). An increase in the concentration of 5-HMF to 0.5, 1, and 2 mM did not result in any significant change compared to the 0.25 mM 5-HMF value. Correlation of the used 5-HMF concentrations with the estimated thiol content resulted in a potential function (y = 33.91x^−0.217^) and a regression term of r = 0.96, showing a saturation of thiols at higher 5-HMF levels.

Comparing the values of VitC and 5-HMF at the same concentrations, a better reduction in the total thiols by 5-HMF is clearly evident, with a significance of *p* < 0.05 at a concentration of 0.25 mM.

This changes with increasing concentrations: while there was no difference in the reduction in thiols at a concentration of 0.5 mM, it was obvious at 1 mM and 2 mM in favor of VitC compared to 5-HMF (1 mM: *p* < 0.01; 2 mM: *p* < 0.001).

### 2.6. Copper Induced Oxidation of LDL Presence or Absence of 5-HMF vs. VitC Measured by TBARS

**Figure 3:**
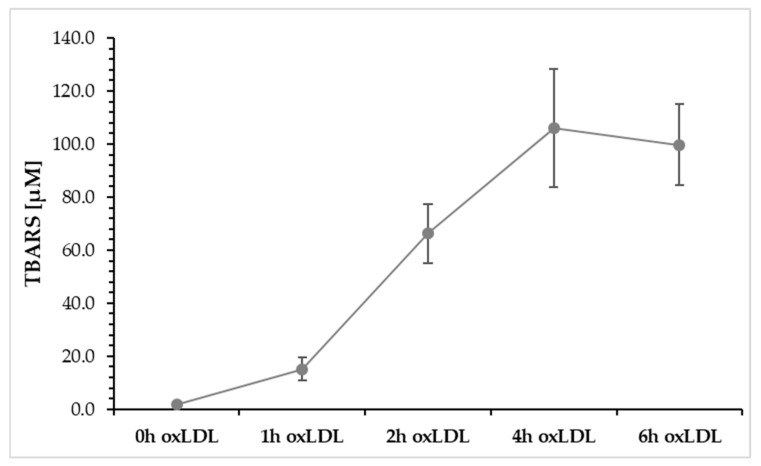
Typical oxidation curve of copper oxidized LDL using TBARS as measure variable (*n* = 3).

Figure 3 describes the oxidation of LDL from 0 to 6 h using a Cu^2+^ solution, measured by TBARS. It shows that after 1 h of oxidation, TBARS increases to a value of 15.2 ± 0.5 nmol/mg. After 2 h, a TBARS value of 66.2 ± 11.2 nmol/mg was measured, and after another 4 h, a concentration of 105.9 ± 22.3 nmol/mg was measured, which represents the maximum value. After 6 h of LDL oxidation, TBARS begins to decline (99.8 ± 15.3 nmol/mg) because TBARS can react with other degradation products, such as aldehydes.

**Figure 4:**
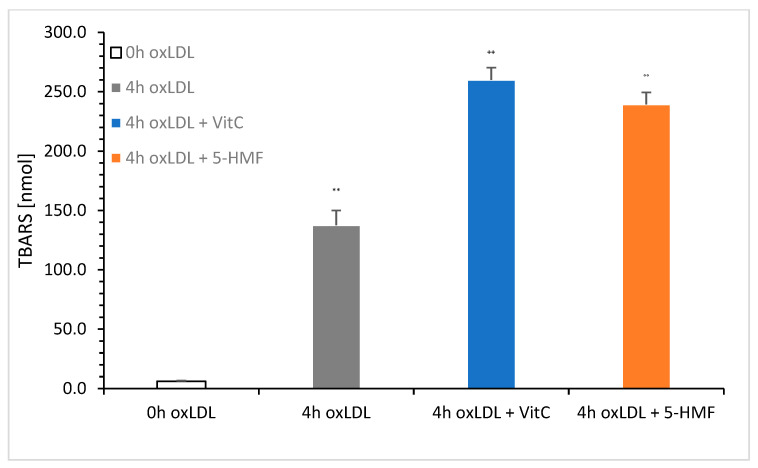
TBARS measurements of 0 h LDL, 4 h oxLDL + 6 mM VitC, or 6 mM 5-HMF after Cu^2+^ mediated oxidation of LDL. ** *p* < 0.001: significance between 0 and 4 h oxLDL; ^++^ *p* < 0.001: significance between 4 h oxLDL + VitC and 0 h oxLDL; °° *p* < 0.001: significance between 4 h oxLDL + 5-HMF and 4 h oxLDL.

For further analysis, we used the 4 h oxLDL value, as this indicates the highest TBARS value. Figure 4 shows a comparison of native LDL (0 h oxLDL), 4 h oxLDL, 4 h oxLDL + VitC, and 4 h oxLDL + 5-HMF. The total concentration was set to 6 mM VitC or 5-HMF in the oxidation solution.

The measurement showed a clear increase in TBARS in the presence of VitC (259.9 ± 10.4 nmol/mg; *n* = 3) and 5-HMF (239.3 ± 10.2 nmol/mg; *n* = 3). There was no significant difference between these two 4 h oxLDLs. Significance was achieved between 4 h oxLDL (137.7 ± 12.3 nmol/mg; *n* = 3) and 4 h oxLDL Vit C (*p* < 0.001) and between 4 h oxLDL and 4 h oxLDL + 5-HMF (*p* < 0.001). The addition of VitC and 5-HMF nearly doubled the TBARS value compared to the 4 h oxLDL baseline value.

## 3. Discussion

There are many natural substances that exhibit reductive behavior, the most important of which are vitamin C and vitamin E. However, what about 5-HMF? There are several methods for measuring its potential. The simplest involves the reduction of metals in complexes, such as the Cu^2+^(BCA)_4_ complex. In the presence of anti-oxidants, the Cu^2+^(BCA)_4_ in the complex is reduced to Cu^+^, which in turn forms a complex with Cu^+^(BCA)_4_. Even proteins exhibit reductive behavior due to their peptide bonds, which has led to this technique being used to determine proteins in blood plasma or biological fluids [16]. However, substances such as vitamin C can interfere with protein determination, particularly at higher concentrations of proteins, as expected [17]. Here, it was clearly shown that small amounts of vitamin C reduced the complex alone. This reductive behavior increased with the concentration of vitamin C. 5-HMF showed lesser potential to reduce the Cu^2+^(BCA)_4_ complex. If free Fe^3+^ is used, it can be reduced to Fe^2+^ by anti-oxidants such as vitamin C. Phenanthroline can then form a complex with the reduced Fe^2+^ to Fe^2+^(phe)_3_. We were able to demonstrate this clearly with vitamin C, but in a weak manner with 5-HMF.

5-HMF showed a property of breaking down peroxynitrite. Peroxynitrite reacted with luminol and induced a chemiluminescence signal in which luminol was oxidized to a dioxetane-ring molecule through an electron transfer. In our experiment highly concentrated VitC broke down almost all the peroxynitrite used, whereas 5-HMF could only do so to a certain extent. At low concentrations 5-HMF was more effective compared to VitC because of different obtained kinetic curves: while VitC catalyzed the reaction with peroxynitrite exponentially, 5-HMF showed a logarithmic form. So, an increase in 5-HMF had limiting effects in reducing peroxynitrite-induced chemiluminescence. The behavior of peroxynitrite was in oxidizing the hydroxyl group of 5-HMF and/or nitrating the furan ring. In addition, 5-HMF is used as an important biomass, which could be converted into several products. Studies were performed in which metal nitrate catalysis converted 5-HMF to 2,5-diformylfuran under an oxygen atmosphere because 5-HMF is used as an important raw material for pharmaceuticals, ligands, or organic conductors [18]. For the production of peroxynitrite, we used the reaction of sodium nitrite and H_2_O_2_ under alkaline conditions. The remaining H_2_O_2_ was reduced with the addition of manganese. It is to be taken into account that under physiological pH at 37 °C, peroxynitrite nitrating reactions are triggered with 5-HMF.

However, it was interesting to note that vitamin C inhibited the nitration of tyrosine residues on BSA just as effectively as 5-HMF. The addition of peroxynitrite to tyrosine in simple phosphate solutions at pH 7.4 resulted in an up to 8% yield of nitro-tyrosine [14]. We used 12 mM ONOO^−^ in our assay, which led to a calculated concentration of 0.96 mM nitro-tyrosine level. Although 5-HMF can break down ONOO^−^ up to a certain concentration, there was enough 5-HMF present (3–6 mM) to almost completely prevent the nitration of tyrosine residues on BSA.

5-HMF has an aldehyde group that can perform nucleophilic addition in alkaline pH ranges. Such a reaction, known as an aldol condensation reaction, could potentially occur with lipids in particular. This should be particularly noticeable with functional groups on lipids and proteins (amines and hydroxyl groups). Combining protein oxidation or lipid oxidation with iron and peroxides, like H_2_O_2_, results in the so-called Fenton reaction (Fe^2+^/H_2_O_2_). It is known that vitamin C can accelerate this reaction. In our experiment, human plasma was mixed with different concentrations of vitamin C and incubated for 90 min. The results showed a higher generation of MDA or HNE with increasing vitamin C levels, while the content of the total thiols in the human plasma decreased in a concentration-dependent manner. 5-HMF appeared to accelerate the production of MDA or HNE more than VitC, even at low concentrations. To date, there has been no indication that 5-HMF can catalyze the production of toxic lipids even higher compared to VitC. With increasing 5-HMF concentrations, this rose even further. A comparison of the total thiol content showed that 5-HMF was also able to reduce thiols, but to a lesser extent compared to vitamin C.

If oxidation is reduced to metals only, Cu^2+^-mediated oxidation of LDL is an approved method for comparing lipid peroxidation using TBARS in the presence of 5-HMF versus VitC. After 4 h of LDL oxidation, both 5-HMF and VitC showed an increase in TBARS production compared to the 4 h oxLDL value or the baseline value. Although it has been reported that vitamin C could delay LDL oxidation [19] and increase lipid hydroperoxides significantly, no data were available about 5-HMF. Here we presented 5-HMF for the first time to increase lipid peroxides during copper-mediated oxidation of LDL. It appeared that 5-HMF in the presence of lipids under slightly alkaline conditions and elevated temperatures led to very complex products, such as modified lipids, degraded 5-HMF, and humins [20], which could not only have side effects like anaphylactoid reactions in vivo and vitro, but also cancerogenic and possibly atherosclerotic effects.

## 4. Materials and Methods

The following materials were purchased: NaNO_2_, FeCl_3_ and CuSO_4_ (Sigma Aldrich, Vienna, Austria), NaOH (Merck, Darmstadt, Germany), MnO_2_, native LDL (Sigma Aldrich, Vienna, Austria), luminol (3-amino-phtalhydrazid, Sigma Aldrich, Vienna, Austria), bovine serum albumin (BSA; Sigma Aldrich, Vienna, Austria), rabbit anti-nitro-tyrosine IgG (Cayman Europe, Tallinn, Estonia), goat anti-rabbit IgG-HRP (Agilent Technologies, Santa Clara, CA, USA), total thiol kit, TMB and stop solution (Immundiagnostik, Bensheim, Germany), Tween 20 (Sigma Aldrich, Vienna, Austria), I-Block (ThermoFisher, Vienna, Austria), 5-HMF and Vitamin C (Sigma Aldrich, Vienna, Austria), lipid peroxidation product (Oxford Biomedical Research, Rochester Hills, MI, USA), CHOD-PAP (Greiner Diagnostic GmbH, Bahlingen, Germany), and human plasma from research donors (London, UK).

### 4.1. Preparation of ONOO^−^ and ONOO^−^-Luminol Measurements Using 5-HMF vs. VitC

ONOO^−^ was produced at low temperature around 0 °C using equal molar amounts of sodium nitrite and hydrogen peroxide solutions [21]. The excess of peroxides was removed by addition of manganese oxide (MnO_2_). After filtration, the concentration was determined spectrophotometrically at 302 nm and used for further measurements.

The degradation of peroxynitrite by 5-HMF was estimated by using a chemiluminescent solution [22] and compared with VitC. According to Greilberger et al. [6], each individual result was determined by averaging the integrals of the curves (BMG Lumistar, Servo LAB, Graz, Austria).

### 4.2. Nitration of BSA with ONOO^−^ in the Presence of 5-HMF vs. VitC

The peroxynitrite-mediated nitration of tyrosine residues fb bovine serum albumin (BSA) was carried out according to the instructions of Greilberger et al. [6].

For the subsequent measurements of nitro-tyrosine residues on BSA, an ELISA method was used in which an antibody specific to nitro-tyrosine residues on BSA was employed [6] using a Power WaveX plate photometer (Bio-Tek Instruments, Winooski, VT, USA).

### 4.3. Estimation of the Reduction Potential of 5-HMF vs. VitC on Cu^2+^ Complexed BCA

For the BCA assay solution 1 (BCA) was mixed 50:1 with solution 2 (Cu^2+^ containing solution). In total, 100, 50, 25, 12.5, 6.25, 3.125, 1.563, 1.782, and 0 mM of 5-HMF or VitC in 100 mM PBS pH 7.4 were prepared for the final mix. A total of 5 µL of each solution was pipetted into the wells of a 96 microtitration plate, and 195 µL of the BCA-Cu^2+^ solution was added into each well, incubated 30 min at 25 °C, and absorption was estimated at λ = 572 nm (Beckmann Coulter DTX Multimode Detector, Servo LAB, Graz, Austria).

### 4.4. Estimation of Reduction Potential of 5-HMF vs. VitC Using Phenanthroline and Fe^3+^

A Fe(III) chloride solution (6 mM) was mixed with different amounts (0.03–3.3 mM) of 5-HMF or VitC to reduce it to Fe(II) for 30 min. Reduced Fe(II) samples were added to a phenanthroline solution (100 mM in water), forming a complex only with Fe(II), but not with Fe(III). The adsorption of the complex was measured at λ = 510 nm (Beckmann Coulter DTX Multimode Detector).

### 4.5. Fenton Reaction of Human Plasma in Presence or Absence of 5-HMF Versus VitC Using Fe^2+^/H_2_O_2_

A total of 500 µL of commercially available human plasma was mixed with 0, 2.5, 5, 7.5, and 10 µL of 1 M HMF versus 1 M VitC. The Fenton reaction was started by adding 3.5 µL of a 1 mg/mL FeCl_3_ solution and 10 µL of 3% H_2_O_2_ solution. After 90 min of incubation, the reaction was stopped by adding 4 µL of a BHT solution in EtOH and 5 µL of a 100 mg/mL EDTA solution. All the samples were analyzed to determine lipid peroxidation products such as MDA, HNE, and HAE. In addition, the total anti-oxidant thiols were estimated.

The MDA or HNE amount in plasma was determined in presence or absence of 5-HMF or VitC using a commercially available test kit (Oxford Biomedical Research, Oxford, USA) [18,19,20] (Beckmann Coulter DTX Multimode Detector, Servo LAB, Graz, Austria).

A commercially available test kit was used to determine the total thiols (Immundiagnostik AG, Bensheim, Germany) on a Beckmann Coulter DTX Multimode Detector (Servo LAB, Graz, Austria).

### 4.6. Cu^2+^ Oxidation of LDL Presence or Absence of 5-HMF vs. VitC Measured by TBARS

A total of 1 mg/mL LDL was prepared in 10 mM PBS, pH 7.4, and carefully degassed. After adding 5 µL of 6 M 5-HMF versus 6 M VitC and 5 µL PBS pH 7.4 as a zero value, oxidation was started with 20 µM Cu^2+^. After 0, 1, 2, 3, and 4 h of incubation, aliquots were taken, stopped with BHT, and stored at a cool temperature until TBARS determination. TBARS of all the samples were estimated using 500 µL of the CHOD-PAP test kit and 50 µL of each LDL aliquot. After 30 min incubation at room temperature, absorbance of all the aliquots was measured at 365 nm (Beckmann Coulter DTX Multimode Detector). The concentrations were calculated according to recent research [23].

### 4.7. Statistics

SPSS statistics (Version 30; SPSS Inc., Chicago, IL, USA) was used to present the appropriate calculations in the specified figures, tables, and result texts. Statistical differences were presented in terms of significance, ranging from *p* < 0.05 as significant to *p* < 0.001 as highly significant. In addition, correlations were determined and presented in curve calculations and regression terms.

## 5. Conclusions

In summary, the results clearly indicate a pro-oxidative property of 5-HMF in metal-induced oxidation of lipids and lipoproteins to generate lipid peroxides. Increased lipid degradation products and lipid radicals are strongly involved not only in the formation of atherosclerotic plaques, a hallmark of atherosclerosis and related diseases, but also in cancer and anaphylaxis.

## Figures and Tables

**Table 1:** Formation of Cu^+^(BCA)_4_ complex in the presence of different VitC or 5-HMF concentrations (*n* = 3), n.s. = no significance.

	Cu^+^(BCA)_4_		
	VitC	5-HMF	
µM	OD [572 nm]	OD [572 nm]	*p*
0	0.099 ± 0.02	0.053 ± 0.02	n.s.
16	0.377 ± 0.015	0.102 ± 0.01	<0.05
31	0.711 ± 0.035	0.116 ± 0.03	<0.001
63	1.242 ± 0.064	0.118 ± 0.027	<0.001
125	2.444 ± 0.184	0.127 ± 0.022	<0.001

**Table 2:** Formation of a Fe^2+^ phenanthroline complex in the presence of different VitC or 5-HMF concentrations (*n* = 3), n.s. = no significance.

	Fe^2+^(phe)_3_		
	VitC	5-HMF	
µM	OD [510 nm]	OD [510 nm]	*p*
0	0.06 ± 0.02	0.06 ± 0.02	n.s.
30	0.199 ± 0.041	0.098 ± 0.024	<0.05
50	0.369 ± 0.035	0.102 ± 0.016	<0.001
100	0.581 ± 0.052	0.118 ± 0.027	<0.001
200	0.888 ± 0.021	0.127 ± 0.022	<0.001

**Table 3:** OD of MDA [586 nm] in the presence or absence of different VitC or 5-HMF concentrations after Fe^2+^/H_2_O_2_ oxidation of human plasma for 90 min at 37 °C (*n* = 3), n.s. = no significance.

	MDA		
	VitC	5-HMF	
mM	OD [586 nm]	OD [586 nm]	*p*
0	0.066 ± 0.002	0.072 ± 0.002	n.s.
0.125	0.072 ± 0.010	0.868 ± 0.017	<0.001
0.25	0.099 ± 0.004	0.939 ± 0.024	<0.001
0.5	0.135 ± 0.009	1.098 ± 0.033	<0.001
1	0.192 ± 0.035	1.209 ± 0.043	<0.001

**Table 4:** OD of HNE [586 nm] in the presence or absence of different VitC or 5-HMF concentrations after Fe^2+^/H_2_O_2_ oxidation of human plasma for 90 min at 37 °C (*n* = 3), n.s. = no significance.

	HNE		
	VitC	5-HMF	
mM	OD [586 nm]	OD [586 nm]	*p*
0	0.037 ± 0.001	0.040 ± 0.001	n.s.
0.125	0.036 ± 0.007	0.609 ± 0.002	<0.001
0.25	0.047 ± 0.001	0.646 ± 0.020	<0.001
0.5	0.077 ± 0.008	0.743 ± 0.027	<0.001
1	0.123 ± 0.034	0.783 ± 0.014	<0.001

**Table 5:** Estimation of the content of thiols in the presence or absence of different VitC or 5-HMF concentrations after Fe^2+^/H_2_O_2_ oxidation of human plasma for 90 min at 37 °C (*n* = 3), n.s. = no significance.

	Thiols		
	VitC	5-HMF	
mM			*p*
0	340.8 ± 14.4 µM	350.4 ± 36.6 µM	n.s.
0.25	312.3 ± 19.7 µM	272.4 ± 4.0 µM	<0.05
0.5	282.4 ± 8.2 µM	259.2 ± 7.8 µM	n.s.
1	198.4 ± 7.7 µM	256.2 ± 7.8 µM	<0.001
2	160.0 ± 13.4 µM	242.4 ± 2.5 µM	<0.001

## Data Availability

The original contributions presented in this study are included in the article. Further inquiries can be directed to the corresponding author(s).

## Abbreviations

The following abbreviations are used in this manuscript:

MDA	Malondialdehyde
HNE	4-Hydroxy-nonenal
TBARS	Thiobarbituric Acid Reacting Substances
VitC	Vitamin C
5-HMF	5-hydroxy-methylfurfural
BCA	Bicinchoninic Acid
BSA	Bovine Serum Albumin
Phe	Phenanthroline

## References

[1] Bachmann S., Meier M., Kanzig A. (1997). 5-Hydroxymethyl-2-furfural (HMF) in Lebensmitteln. Lebensmittelchemie.

[2] Shapla U.M., Solayman M., Alam N., Khalil M.I., Gan S.H. (2018). 5-Hydroxymethylfurfural (HMF) levels in honey and other food products: Effects on bees and human health. Chem. Cent. J..

[3] EFSA (2011). Scientific Opinion on the re-evaluation of caramel colours (E 150 a,b,c,d) as food additives. EFSA Panel on Food Additives and Nutrient Sources added to Food (ANS); Scientific Opinion on the re-evaluation of caramel colours (E 150a, b,c, d) as food additives. EFSA J..

[4] Bauer-Marinovic M., Taugner F., Florian S., Glatt H. (2012). Toxicity studies with 5-hydroxymethylfurfural and its metabolite 5-sulphooxymethylfurfural in wild-type mice and transgenic mice expressing human sulphotransferases 1A1 and 1A2. Arch. Toxicol..

[5] Monien B.H., Frank H., Seidel A., Glatt H.R. (2009). Conversion of the common food constituent, 5-hydroxymethylfurfural, into a mutagenic and carcinogenic sulphuric acid ester in the mouse in vivo. Chem. Res. Toxicol..

[6] Greilberger J., Herwig R., Greilberger M., Stiegler P., Wintersteiger R. (2021). Alpha-Ketoglutarate and 5-HMF: A Potential Anti-Tumoral Combination against Leukemia Cells. Antioxidants.

[7] Pagare P.P., McGinn M., Ghatge M.S., Shekhar V., Alhashimi R.T., Daniel Pierce B., Abdulmalik O., Zhang Y., Safo M.K. (2024). The antisickling agent, 5-hydroxymethyl-2-furfural: Other potential pharmacological applications. Med. Res. Rev..

[8] Qiu Y., Lin X., Chen Z., Li B., Zhang Y. (2022). 5-Hydroxymethylfurfural Exerts Negative Effects on Gastric Mucosal Epithelial Cells by Inducing Oxidative Stress, Apoptosis, and Tight Junction Disruption. J. Agric. Food Chem..

[9] Jiang Y., Geng N., Wang M., Wu W., Feng N., Zhang X. (2022). 5-HMF affects cardiovascular development in zebrafish larvae via reactive oxygen species and Wnt signaling pathways. Comp. Biochem. Physiol. C Toxicol. Pharmacol..

[10] Chen X., Tu Q., Zhao W., Lin X., Chen Z., Li B., Zhang Y. (2024). 5-Hydroxymethylfurfural mediated developmental toxicity in Drosophila melanogaster. Food Chem. Toxicol..

[11] Kong F., Lee B.H., Wei K. (2019). 5-Hydroxymethylfurfural Mitigates Lipopolysaccharide-Stimulated Inflammation via Suppression of MAPK, NF-κB and mTOR Activation in RAW 264.7 Cells. Molecules.

[12] Zhao L., Chen J., Su J., Li L., Hu S., Li B., Zhang X., Xu Z., Chen T. (2013). In vitro antioxidant and antiproliferative activities of 5-hydroxymethylfurfural. J. Agric. Food Chem..

[13] Timoshnikov V.A., Kobzeva T.V., Polyakov N.E., Kontoghiorghes G.J. (2020). Redox Interactions of Vitamin C and Iron: Inhibition of the Pro-Oxidant Activity by Deferiprone. Int. J. Mol. Sci..

[14] Beckman J.S. (1996). Oxidative damage and tyrosine nitration from peroxynitrite. Chem. Res. Toxicol..

[15] Greilberger J., Herwig R., Kacar M., Brajshori N., Feigl G., Stiegler P., Wintersteiger R. (2022). Alpha-Ketoglutarate or 5-HMF: Single Compounds Effectively Eliminate Leukemia Cells via Caspase-3 Apoptosis and Antioxidative Pathways. Int. J. Mol. Sci..

[16] Kapoor K.N., Barry D.T., Rees R.C., Dodi I.A., McArdle S.E., Creaser C.S., Bonner P.L. (2009). Estimation of peptide concentration by a modified bicinchoninic acid assay. Anal. Biochem..

[17] Jampel H.D. (1994). Determination of protein concentration in aqueous humor. J. Glaucoma.

[18] Hong M., Min J., Wu S., Cui H., Zhao Y., Li J., Wang S. (2019). Metal Nitrate Catalysis for Selective Oxidation of 5-Hydroxymethylfurfural into 2,5-Diformylfuran under Oxygen Atmosphere. ACS Omega.

[19] Suh J., Zhu B.Z., Frei B. (2003). Ascorbate does not act as a pro-oxidant towards lipids and proteins in human plasma exposed to redox-active transition metal ions and hydrogen peroxide. Free Radic. Biol. Med..

[20] Li E., Lin N., Hao R., Fan X., Lin L., Hu G., Lin S., He J., Zhu Q., Jin H. (2020). 5-HMF induces anaphylactoid reactions in vivo and in vitro. Toxicol. Rep..

[21] Hughes M.N., Nicklin H.G. (1968). The chemistry of pernitrites. Part I. Kinetics of decomposition of pernitrous acid. J. Chem. Soc. A.

[22] Radi R., Cosgrove T.P., Beckman J.S., Freeman B.A. (1993). Peroxynitrite-induced luminol chemiluminescence. Biochem. J..

[23] Esterbauer H., Schaur R.J., Zollner H. (1991). Chemistry and biochemistry of4-hydroxynonenal, malonaldehyde and related aldehydes. Free Radic. Biol. Med..

